# Women and children living in areas of armed conflict in Africa: a geospatial analysis of mortality and orphanhood

**DOI:** 10.1016/S2214-109X(19)30407-3

**Published:** 2019-10-24

**Authors:** Zachary Wagner, Sam Heft-Neal, Paul H Wise, Robert E Black, Marshall Burke, Ties Boerma, Zulfiqar A Bhutta, Eran Bendavid

**Affiliations:** a RAND Corporation, Santa Monica, CA, USA; b Center on Food Security and the Environment, Stanford University, Stanford, CA, USA; c The Center for Health Policy and the Center for Primary Care and Outcomes Research, Stanford University, Stanford, CA, USA; d Department of Pediatrics, Stanford University, Stanford, CA, USA; e Department of Earth System Science, Stanford University, Stanford, CA, USA; f Center for Population Health Sciences, Division of Primary Care and Population Health, Department of Medicine, Stanford University, Stanford, CA, USA; g The Institute for International Programs, Bloomberg School of Public Health, John Hopkins University, Baltimore, MD, USA; h Center for Global Public Health, University of Manitoba, Winnipeg, MB, Canada; i Centre for Global Child Health, Hospital for Sick Children (SickKids), Toronto, ON, Canada; j The Center of Excellence in Women and Child Health, Aga Khan University, Karachi, Pakistan

## Abstract

**Background:**

The population effects of armed conflict on non-combatant vulnerable populations are incompletely understood. We aimed to study the effects of conflict on mortality among women of childbearing age (15–49 years) and on orphanhood among children younger than 15 years in Africa.

**Methods:**

We tested the extent to which mortality among women aged 15–49 years, and orphanhood among children younger than 15 years, increased in response to nearby armed conflict in Africa. Data on location, timing, and intensity of armed conflicts were obtained from the Uppsala Conflict Data Program, and data on the location, timing, and outcomes of women and children from Demographic and Health Surveys done in 35 African countries from 1990 to 2016. Mortality among women was obtained from sibling survival data. We used cluster-area fixed-effects regression models to compare survival of women during periods of nearby conflict (within 50 km) to survival of women in the same area during times without conflict. We used similar methods to examine the extent to which children living near armed conflicts are at increased risk of becoming orphans. We examined the effects of varying conflict intensity using number of direct battle deaths and duration of consecutive conflict exposure.

**Findings:**

We analysed data on 1 629 352 women (19 286 387 person-years), of which 103 011 (6·3%) died (534·1 deaths per 100 000 women-years), and 2 354 041 children younger than 15 years, of which 204 276 (8·7%) had lost a parent. On average, conflict within 50 km increased women's mortality by 112 deaths per 100 000 person-years (95% CI 97–128; a 21% increase above baseline), and the probability that a child has lost at least one parent by 6·0% (95% CI 3–8). This effect was driven by high-intensity conflicts: exposure to the highest (tenth) decile conflict in terms of conflict-related deaths increased the probability of female mortality by 202% (187–218) and increased the likelihood of orphanhood by 42% compared with a conflict-free period. Among the conflict-attributed deaths, 10% were due to maternal mortality.

**Interpretation:**

African women of childbearing age are at a substantially increased risk of death from nearby high-intensity armed conflicts. Children exposed to conflict are analogously at increased risk of becoming orphans. This work fills gaps in literature on the harmful effects of armed conflict on non-combatants and highlights the need for humanitarian interventions to protect vulnerable populations.

**Funding:**

Bill & Melinda Gates Foundation to the BRANCH Consortium.

## Introduction

Between 40 and 68 countries, home to 46–79% of the world's population, were involved in armed conflict in every year since 1990.^1^, ^2^, ^3^ Contrary to claims that wars, including armed conflicts, have been steadily receding since the mid 1940s,^4^ there is little evidence of a decline in the number of conflicts, the number of countries involved in conflict, or the proportion of the world's population living in conflict-affected regions since 1990.^2^, ^5^ The direct consequences of armed conflict are readily visible and include destruction of physical environments, injuries, and deaths of combatants and uninvolved civilians. However, the toll of armed conflicts on vulnerable populations—excess mortality and morbidity from non-violent causes—is often not shown in battlefield images and underappreciated in body counts.^6^, ^7^, ^8^ Attempts to quantify a more complete picture of mortality due to conflict, including those in Iraq and South Sudan, suggest that the total number of deaths attributable to armed conflicts typically far exceed the estimates of direct conflict deaths.^9^, ^10^, ^11^

We estimated that the number of children under 1 year and under 5 years whose deaths can be attributed to armed conflict was three to five times greater than the number of people that died through direct involvement in armed conflicts across Africa between 1995 and 2015.^12^

The lives of women of childbearing age might be endangered in areas of armed conflict for several reasons. In DR Congo, where chronic conflict was implicated as a reason for the 40% higher crude mortality relative to the regional average, women are raped and used as weapons of war.^13^, ^14^ The hazards of childbirth might also be increased in areas of conflict, because basic services, such as facilities for safe labour and delivery that reduce the mortality from post-partum haemorrhage or stalled labour, might not be accessible.^15^ Conflict-related destruction of basic family and social structures, including households and local communities, leave women exposed and potentially vulnerable to risks, especially during pregnancy or while caring for young children.^16^ In our analysis of conflict's risks to young children, we noted that neonatal mortality is increased even when the conflict occurred during the year before birth, which is suggestive of harms borne by the neonates' mothers.^12^

**Research in context**

**Evidence before this study**

The population effects of armed conflict on non-combatant vulnerable populations, especially women and children, are incompletely understood. Armed conflict leads to substantial increases in the mortality risk of children in Africa. In addition, child mortality improvements lag during periods of conflict. The non-fatal effects of conflict on children include moderate increases in stunting and delays in schooling. However, little is known about the dangers of armed conflict for mortality of women of childbearing age, who are rarely involved in the fighting. Moreover, the extent to which conflict leaves surviving children as orphans is unknown. Although we did not do a formal literature search, the evidence we identified that documented the consequences of conflict on non-combatants generally examines one conflict or one geographic setting (eg, Rwanda, Burundi, or the Ethiopia–Eritrea conflicts). The extent of the burden of armed conflict on women's mortality and orphanhood in Africa has not been previously quantified.

**Added value of this study**

Our study provides new insights and new breadth about the harmful effects of armed conflict on mortality among women of childbearing age and the probability of orphanhood. First, the scope of our study is far broader than previous work. We analysed over 15 000 conflict events across 33 African countries from 1990 to 2016. These events included 1 629 352 women (19 286 387 women-years) and 103 011 deaths. Second, we identified a 21% increase in mortality for women who lived within 50 km of an armed conflict event compared with baseline (ie, no conflict). This increase was mostly driven by very intense and deadly conflict events. Third, we showed that 10% of all conflict-attributable deaths for women were due to maternal mortality. These deaths are probably caused by indirect effects, such as deteriorated health infrastructure, rather than the violence itself. Finally, we showed that children 0–15 years-old exposed to armed conflict were 6% more likely to be orphans when living near any conflict (on average), and 42% more likely when living near conflicts of the highest intensity.

**Implications of all the available evidence**

This work brings new evidence on the harmful effects of armed conflict on non-combatant populations and highlights the need for developing effective humanitarian interventions to protect these vulnerable populations. We also highlight the distinctions between types of conflicts during which the risks to women and orphans are small and those during which the risks are high.

Conflict's effects on women of childbearing age might, in turn, have additional effects on their children. Conflicts during which women are at a substantially high risk of dying can have important implications in the number of orphans and the fate of children (children might be orphaned by the loss of their mother or father; in this Article, we focus on women and children). Conflict is not the only cause of orphanhood; parents of young children can die from several reasons and HIV has played a big role in African orphanhood.^17^, ^18^ However, if the risk of orphanhood increases with conflict, it could have important long-term effects on human capital and the health of affected populations.^19^, ^20^, ^21^ Responses to conflict, both anticipatory preparation of services before and after conflict mitigation of health consequences, could be improved by better understanding these patterns.

In this Article, we examined the effects of armed conflicts on mortality among women of childbearing age and orphanhood. We estimated the extent to which the risk of death among women aged 15–49 years is increased following a nearby armed conflict relative to their expected risk of dying. These deaths might be due to either direct or indirect effects of conflict, and we used available information on cause and timing of death to assess the extent to which direct and indirect conflict effects are involved. For children younger than 15 years, we estimated the effect of conflict on orphanhood as the probability that one or both of the child's parents are reported as deceased subsequent to nearby conflict events.

## Methods

### Data sources

Our primary data source on armed conflict was the Uppsala Conflict Data Program Georeferenced Events Dataset (UCDP GED).^1^, ^22^ The dataset includes the time, location, type, and intensity of conflict events, with geocoded location events, from Jan 1, 1989, to Dec 31, 2017. A conflict event is defined in the UCDP GED as “the incidence of the use of armed force by an organised actor against another organised actor, or against civilians, resulting in at least one direct death”.^22^ The UCDP uses news sources, non-governmental organisation reports, case studies, truth commission reports, historical archives, and other sources of information to select, categorise, and localise conflict events. We used all conflict events in the UCDP dataset with at least one conflict-related death in any African country from 1989 to 2017 to estimate exposure to conflict.

Information on orphans and mortality among women of childbearing age came from the Demographic and Health Surveys (DHS).^23^, ^24^ The DHS are nationally representative surveys done in most African countries. The surveys include household census modules that contain information about the vital status of the parents of all children residing in the household (which we use to identify orphans) and sibling survival modules that record the vital status of all the index respondent's siblings (which we use to identify mortality among women of childbearing age; index respondents are women aged 15–49 years).^25^ Most DHS in Africa also contain latitude–longitude coordinates of the survey cluster (equivalent to a village or neighbourhood), displaced by 2–5 km for privacy. We used data from every African DHS with geospatial identifiers and information on sibling mortality or orphanhood.^26^ Because the UCDP contains a complete register of (detectable) armed conflicts, we can match every DHS respondent to all relevant armed conflict exposures.

### Definitions of conflict exposure, women's mortality, and orphanhood

We determined the exposure of each household to conflict by geospatially and temporally linking the household location and individual outcomes to nearby conflict events. We used two related primary measures of exposure: whether or not an armed conflict event resulting in direct conflict-related deaths occurred within 50 km of the household in the calendar year (binary conflict exposure); and the number of conflict-related deaths within 50 km in the calendar year (continuous conflict exposure). We used conflict chronicity as an additional measure of intensity of conflict exposure, which we define as the number of consecutive years a household is exposed to nearby armed conflict. We also measured exposure to an armed conflict event within 51–100 km.

We identified mortality among women of childbearing age using the sibling survival module, in which the index women were asked about the vital status of her siblings from the same mother.^27^ We focus on women of childbearing age because data on older siblings, although recorded in DHS, are sparse and fall outside the scope of this analysis. Each sister's age (we use sisters to refer to siblings who are women of childbearing age), vital status, and age at death (if relevant) were recorded, as well as indicators of deaths that occurred around pregnancy, childbirth, or the first 2 months after delivery (although, strictly speaking, DHS reports on pregnancy-related mortality, these deaths are commonly referred to as maternal deaths and thus we follow this convention).^28^ We use this analysis to create a longitudinal record for each sister with an indicator of whether or not she was alive or dead at the end of each year. The woman-year indicator was then used as our primary outcome indicator for analysing women of childbearing age.

In another part of the DHS, a complete register of all children living in the household includes information about whether or not the child's father or mother had died at the time of the survey. We identify orphans as children younger than 15 years (older children are surveyed as adults in DHS) who had lost either their father or mother, or both. Because the year of parental death is not recorded in the DHS, we define conflict exposure for children as the average annual number of nearby combat deaths that the child had been exposed to at the time of the survey.

### Statistical analysis

The primary statistical approach to identify the effects of conflict on mortality among women of childbearing age and orphanhood follows a conceptual model in which nearby conflict increases the risks of undesirable outcomes above the expected baseline in the same region without conflict. We operationalise this conceptual approach using the following linear probability models:

(1) $$Women'sdeath_{ilct}=\beta_{1}D_{lct}+\rho X_{ilct}+\eta_{lc}+\gamma_{t}+{ɛ}_{ilct}$$

(2) $$Women'sdeath_{ilct}=\sum_{q=1}^{10}\beta_{q}D_{lct}^{q}+\rho X_{ilct}+\eta_{lc}+\gamma_{t}+{ɛ}_{ilct}$$

(3) $$Orphan_{ilcat}=\beta_{1}D_{lcat}+\rho X_{ilcat}+\phi_{a}+\eta_{lc}+\gamma_{t}+{ɛ}_{ilcat}$$

(4) $$Orphan_{ilcat}=\sum_{q=1}^{10}\beta_{q}D_{lcat}^{q}+\rho X_{ilcat}+\phi_{a}+\eta_{lc}+\gamma_{t}+{ɛ}_{ilcat}$$

where the outcomes, indicators that equal 1 if the woman died or if the child was an orphan, are indexed for person *i*, DHS cluster *l*, country *c*, and calendar year of observation *t*; orphans are additionally indexed by age *a*. The vector *X* represents control variables we used to improve the precision of our estimates, including age and educational attainment of the index sibling (for women) or household head (for orphans); to flexibly control for orphans, we included age indicators, represented as φ_a_, to control for the child's age. γ_t_ and η_lc_ are year and cluster fixed effects, respectively, which account for all shared time effects (year-fixed effects) and time-invariant between-cluster differences (cluster-fixed effects), to control for fixed differences between conflict-exposed and conflict-unexposed areas.

Our primary predictors, *D*, are either an indicator for our binary exposure to any armed conflict or indicators for decile of exposure by conflict intensity (in the same year of observation for women and average annual exposure for children). The parameters of interest, β, represent the increase in the probability of the person *i* experiencing the outcome following armed conflicts within 50 km. We cluster our standard errors at the level of the DHS cluster throughout, because it is the primary level of variation for the conflict exposure.^29^ This approach accounts for the fact that observations within each cluster are correlated. Estimating within-cluster and within-year effects (by using the fixed effects η_lc_ and γ_t_) allows us to relax many concerns about between-cluster differences (ie, areas with conflict are fundamentally different from conflict-free areas) and shared trends in the outcomes.

We used our regression results to estimate the excess number and geographical distribution of deaths among women of childbearing age related to conflict in our study countries from 2000 to 2017 (the years for which population estimates were available).^30^ We estimated conflict exposure for each 10 km by 10 km grid cell each year. We then applied the estimated increase in mortality (based on exposure intensity parameter estimates) to the number of women of childbearing age living in each grid cell in each year and summing the estimated number of deaths from 2000 to 2017.

We did multiple supplementary analyses to test hypotheses about mechanisms and address limitations in our data. The surveys contain information on one cause of death—maternal mortality—and we tested the effects of armed conflict using maternal mortality as the outcome. We also tested the robustness of our data to the choice of fixed effects and to country outliers (appendix p 2). We then focused on the potential bias from population displacement (resulting in unobserved refugees or internally displaced people). We evaluated the effects of displacement by doing simulations that bound the implications of displacement on our main outcomes. We simulated displaced individuals and added them into each conflict-affected cluster-year cohort in our data, with their size and vital status based on assumptions about the displacement and mortality among displaced populations relative to non-displaced populations. We then re-estimated our main regression model with the new data. We repeated this process for a range of displacement proportions (10–50%, in 10% steps, of resident population displaced by conflict) and relative mortality (−50% to +50% mortality relative to non-displaced population, informed by studies showing both lower and higher mortality among refugees relative to non-displaced populations; appendix p 5).^31^, ^32^ These ranges are likely to contain the average displacement rate and the average relative mortality rate. Therefore, the range of estimates produced by this analysis is likely to contain the true average treatment effect as if the displaced population was observed. We also show additional analyses on the lasting effect of conflict beyond the contemporaneous effect (appendix p 11), on maternal mortality (appendix p 12), orphanhood by parent (mother or father; appendix p 13), and over distances greater than 50 km (appendix p 14).

The extent to which some groups might be more or less vulnerable to the effects of conflict can help with the design of mitigation strategies. We examined differences in the effect of conflict by wealth quintile (a 5-point relative wealth constructed in each survey from household assets), education of household head,^33^ and place of residence (urban or rural).

### Role of the funding source

The funder of this study had no role in the study design, data collection, data analysis, data interpretation, or writing of the report. The corresponding author had full access to the data in the study and had final responsibility for the decision to submit for publication.

## Results

The samples used for this analysis include all sisters and children younger than 15 years documented in surveys with the minimally suitable data for their respective analyses: sibling survival module for women, household census for orphans, and geospatial coordinates for both. Our analysis includes 73 surveys from 33 countries for women's mortality and 94 surveys from 35 countries for orphanhood (sibling survival modules are not available in all surveys; table 1).

**Table 1:** Demographic and Health Surveys of women and orphans used in this study

	**Women**			**Orphans**	
	Number of women	Women-years	Deaths	Number of children	Orphans
Angola (2015)	21 532	291 164	879	37 234	2740
Benin (1996)	8862	47 771	162	13 339	896
Benin (2001)	..	..	..	14 391	941
Benin (2011)	..	..	..	42 712	2233
Burkina Faso (1992)	..	..	..	15 860	1309
Burkina Faso (1998)	10 097	72 461	346	..	..
Burkina Faso (2003)	20 772	201 928	876	28 629	2125
Burkina Faso (2010)	31 667	396 787	1585	39 786	1993
Burundi (2010)	17 746	208 814	1259	19 303	2111
Burundi (2016)	34 245	472 449	2068	37 151	2915
Cameroon (1991)	..	..	..	9 460	651
Cameroon (2004)	20 839	212 266	1133	22 523	2052
Cameroon (2011)	31 113	402 150	2269	32 143	2715
Central African Republic (1994)	9 726	39 336	336	12 484	1368
Chad (2014)	34 461	464 594	1904	53 832	3515
Comoros (2012)	10 852	142 247	204	9 684	407
Côte d'Ivoire (1994)	13 576	50 484	239	17 558	1118
Côte d'Ivoire (2011)	20 538	273 551	1522	22 267	1711
DR Congo (2007)	19 390	227 984	1277	22 606	1902
DR Congo (2013)	35 934	480 599	2430	48 553	3962
Egypt (2000)	..	..	..	33 569	1607
Egypt (2005)	..	..	..	38 190	1556
Egypt (2008)	..	..	..	31 125	1152
Egypt (2014)	..	..	..	41 270	1232
Ethiopia (2000)	25 235	203 408	1438	29 298	3683
Ethiopia (2005)	24 168	256 428	1638	30 109	3176
Ethiopia (2011)	29 624	371 824	2062	34 830	2967
Ethiopia (2016)	28 242	397 649	1517	33 977	2463
Gabon (2012)	17 362	241 115	963	16 682	870
Ghana (1993)	..	..	..	10 426	746
Ghana (1998)	..	..	..	9 697	610
Ghana (2003)	..	..	..	11 684	788
Ghana (2008)	..	..	..	18 945	1301
Ghana (2014)	..	..	..	18 241	1352
Guinea (1999)	9994	76 386	265	15 828	1310
Guinea (2005)	12 616	142 235	702	18 164	1350
Guinea (2012)	14 577	191 455	879	21 424	1815
Kenya (2003)	18 879	188 357	978	16 158	1804
Kenya (2008)	18 167	229 134	1056	..	..
Kenya (2014)	32 969	483 942	1898	68 662	6381
Lesotho (2004)	11 744	121 620	995	14 805	3857
Lesotho (2009)	12 340	152 442	1439	15 701	3941
Lesotho (2014)	10 246	142 168	1397	13 929	3276
Liberia (2006)	11 978	145 134	596	16 061	1094
Liberia (2013)	17 789	256 074	1063	22 498	1460
Madagascar (1997)	14 910	91 625	430	15 169	1236
Madagascar (2008)	37 142	477 215	1811	38 728	2493
Malawi (2000)	23 320	184 584	1830	29 271	3484
Malawi (2004)	20 429	205 829	1958	29 197	3859
Malawi (2010)	43 695	542 480	4646	58 033	6361
Malawi (2015)	44 317	605 876	3489	56 697	5722
Mali (1995)	15 245	78 597	336	24 003	1464
Mali (2001)	21 114	185 894	901	32 075	1773
Mali (2006)	24 750	272 565	1146	35 200	1901
Mali (2012)	15 266	198 127	454	29 157	1210
Morocco (2003)	38 297	404 758	470	..	..
Mozambique (2009)	..	..	..	12 601	1506
Mozambique (2011)	21 871	289 476	1389	29 157	3435
Mozambique (2015)	..	..	..	15 884	1766
Namibia (2000)	13 673	113 193	441	12 291	1294
Namibia (2006)	20 323	243 375	1685	16 211	2399
Namibia (2013)	17 836	255 737	1443	15 516	1808
Niger (1992)	10 213	20 011	65	16 033	1145
Niger (1998)	..	..	..	17 378	1078
Nigeria (2003)	..	..	..	14 961	1052
Nigeria (2008)	59 626	711 633	2837	69 511	3718
Nigeria (2013)	73 172	1 028 093	3071	79 345	4201
Rwanda (2005)	23 594	243 898	3143	21 857	3866
Rwanda (2010)	28 518	357 719	2951	24 899	2630
Rwanda (2014)	27 445	389 486	2310	23 466	1789
Senegal (1992)	9 906	26 070	72	14 565	933
Senegal (2005)	28 064	303 257	801	30 679	2278
Senegal (2010)	28 259	358 549	984	34 754	2109
Sierra Leone (2008)	10 063	124 151	572	19 528	2064
Sierra Leone (2013)	24 794	332 614	1617	34 148	3070
eSwatini (2006)	9362	104 431	1021	9339	1912
Tanzania (1999)	..	..	..	8586	719
Tanzania (2003)	..	..	..	15 302	1381
Tanzania (2007)	..	..	..	20 647	1695
Tanzania (2009)	21 314	282 937	1113	23 061	1710
Tanzania (2011)	..	..	..	25 209	1952
Tanzania (2015)	27 199	396 173	1652	29 612	2008
Togo (1998)	15 669	108 458	370	20 880	1962
Togo (2013)	18 365	267 016	1019	21 487	1765
Uganda (2000)	14 939	124 784	1090	18 584	2551
Uganda (2006)	17 919	198 520	1656	23 439	3187
Uganda (2011)	18 143	228 375	1543	38 176	4021
Uganda (2016)	38 194	517 914	2704	44 343	3596
Zambia (2007)	14 821	171 740	1888	17 241	2244
Zambia (2013)	34 510	474 234	3950	40 554	4107
Zimbabwe (1999)	12 346	92 967	674	12 218	1743
Zimbabwe (2005)	18 383	199 166	1940	18 586	4045
Zimbabwe (2010)	17 299	227 314	1982	17 518	3349
Zimbabwe (2015)	17 767	263 590	2182	18 157	2230
Total	1 629 352	19 286 387	103 011	2 354 041	204 276

All data are aggregated from Demographic and Health Surveys (94 surveys) done in 35 African countries between 1991 and 2016. Women's mortality data are extracted from sibling survival modules and orphan data from the household census modules.

**Figure 1:**
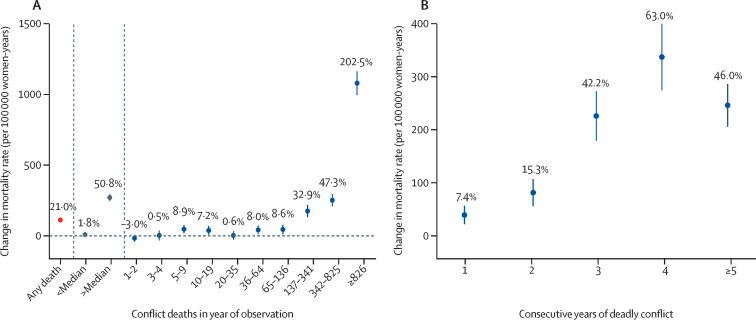
Change in the risk of death for women of childbearing age as a function of proximity to conflict A) Change in average mortality rate due to conflict during year of observation; conflicts are designated as a binary exposure (any death), above median intensity (>35 deaths), or as deciles of exposure intensity by the number of direct combat-related deaths within 50 km. B) Change in average mortality rate due to conflict depending on the number of consecutive years the index woman's cluster of residence has been exposed to nearby conflict (50 km). Error bars indicate 95% CIs.

Figure 1 shows the pattern of mortality among women of childbearing age in response to conflict. Overall, 534·1 deaths occurred for every 100 000 women-years in our sample (table 2). On average, conflict within 50 km increased women's mortality by 112 deaths per 100 000 person-years (95% CI 97–128). This increase represents a 21·0% (95% CI 18·2–23·9) higher risk of death when exposed to conflict relative to their expected mortality risk in the absence of conflict. The increased risk of death was highly dependent on the intensity of exposure: mortality among women was not affected (2% [–1 to 5]) by nearby conflicts when the conflict was below median intensity in terms of conflict-related deaths (fewer than 36 nearby conflict-related deaths in the year of exposure). Above the fifth decile of intensity (36 or more conflict-related deaths), mortality risk increased substantially (51% [46–56]), and at the top decile of conflict intensity (more than 826 nearby conflict-related deaths), the risk of death among women was triple (202% [187–218] higher) of what would be expected in the absence of armed conflict. Women from 12 countries were represented in the highest decile, most commonly from Burundi, Côte d'Ivoire, Liberia, Rwanda, DR Congo, Ethiopia, and Sierra Leone. Figure 1 shows that the relationship with intensity is similarly observed with the duration of conflict (ie, the number of consecutive years a woman is exposed to conflict within 50 km): mortality is increased by 7·4–15·3% for women exposed to conflict for no more than 2 years, but by more than 42% for women exposed to conflict for 3 years or longer.

**Table 2:** Women and children sample summary by conflict exposure

		**Women**				**Children**		
		N	Woman-years	Deaths	Mortality rate (per 100 000 woman-years)	N	Orphans (either parent)	Orphans (both parents)
Total		1 629 352	19 286 387	103 011	534·1	2 354 041	204 276 (8·7%)	24 826 (1·1%)
Not exposed to conflict		984 564	16 463 214	86 109	523·0	1 360 508	112 741 (8·3%)	14 510 (1·1%)
Exposed to conflict		644 788	2 823 173	16 902	598·7	993 533	91 535 (9·2%)	10 316 (1·1%)
Decile of exposure								
	First	63 713	410 441	1843	449·0	95 354	10 806 (11·3%)	1444 (1·5%)
	Second	47 418	224 039	1142	509·7	93 977	9770 (10·4%)	1069 (1·1%)
	Third	52 061	301 452	1464	485·6	105 491	8090 (7·7%)	750 (0·7%)
	Fourth	48 823	290 946	1388	477·1	92 478	8276 (8·9%)	897 (1·0%)
	Fifth	58 875	280 089	1317	470·2	104 842	8860 (8·5%)	838 (0·8%)
	Sixth	70 778	272 484	1321	484·8	99 549	9043 (9·1%)	984 (1·0%)
	Seventh	66 916	275 950	1361	493·2	98 183	8430 (8·6%)	899 (0·9%)
	Eighth	66 215	278 189	1609	578·4	110 449	8846 (8·0%)	865 (0·8%)
	Ninth	53 372	238 731	1588	665·2	100 975	8645 (8·6%)	978 (1·0%)
	Tenth	53 372	250 852	3869	1 542·3	92 235	10 769 (11·7%)	1592 (1·6%)

Data are n or n (%), unless otherwise stated. Women's mortality and orphanhood rates shown in the table are raw (unadjusted) ratios of events divided by exposure. They, therefore, differ from the adjusted estimates shown in figure 3, and do not account for fixed differences between areas exposed to different levels of conflict.

The most direct information related to cause of death of women of childbearing age that is available in the sibling survival data is maternal mortality. We used maternal deaths as the dependent outcome in a regression framework and observed that armed conflict increases maternal deaths by 11% (95% CI 5–17; appendix p 12) overall. The increase in maternal deaths accounted for 10% of all excess deaths among women of childbearing age, whereas maternal deaths make up 19% of all deaths among women of childbearing age in our sample. This result implies that the increases in cause-specific mortality that drive our observed all-cause mortality increase are more pronounced for some non-maternal causes (including violence-related deaths) than for maternal causes.

**Figure 2:**
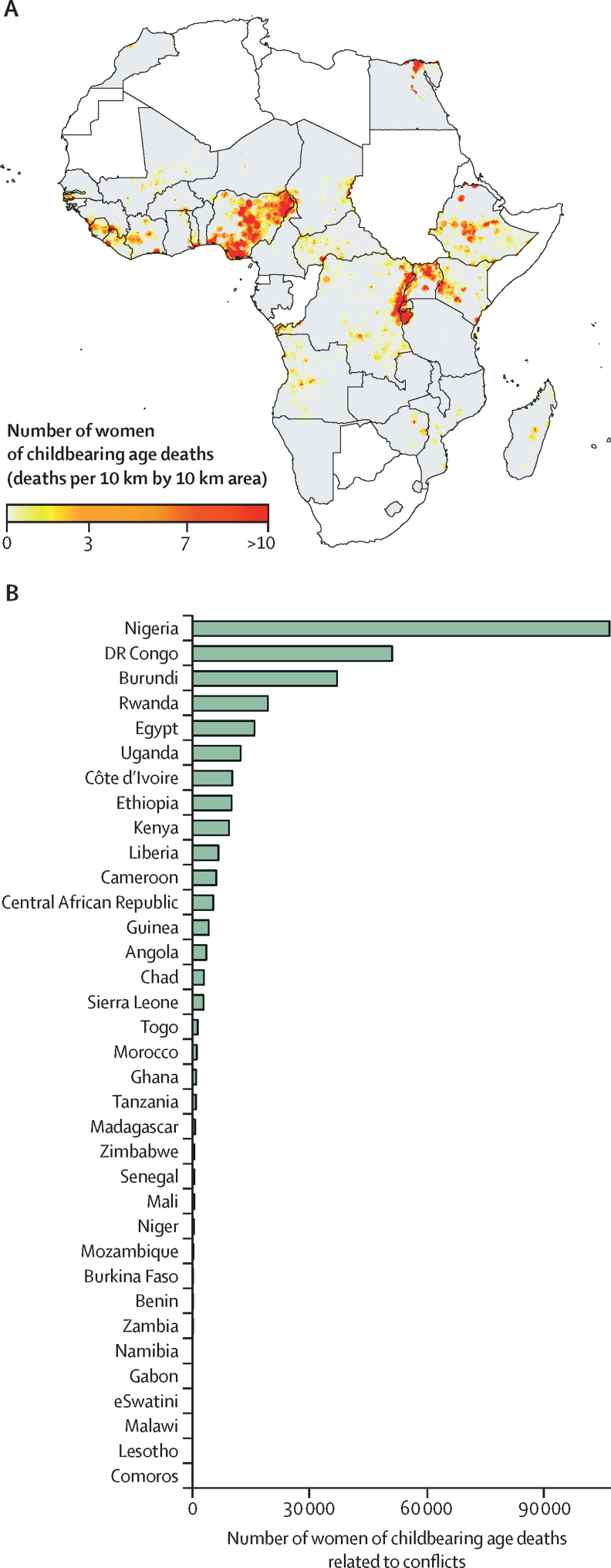
Spatial distribution and estimated number of maternal deaths in each study country (2000–17) (A) Spatial distribution of maternal deaths.^30^ (B) Total deaths by country.

Using our regression parameters combined with UCDP conflict and women of childbearing age population data, we estimated that 310 494 women of childbearing age (95% CI 193 859–478 580) died in the countries included in our study between 2000 and 2017. Nigeria (106 819 deaths) and the DR Congo (51 118 deaths) accrued the largest number of women's deaths related to conflict over this period (figure 2), accounting for about 51% all deaths. If we assume that the average effect sizes are similar in African countries where we did not have population outcome data, then the estimated number of women who died due to conflict between 2000 and 2017 was 426 558 (95% CI 269 721–649 114; about 1·7% of all deaths among women of childbearing age in Africa during this period). Somalia, Sudan, Eritrea, and Libya are the most conflict-intense countries not included in our effect estimation, but there is little evidence to inform us how the effects of armed conflicts on women in these countries might be different from the study countries.

**Figure 3:**
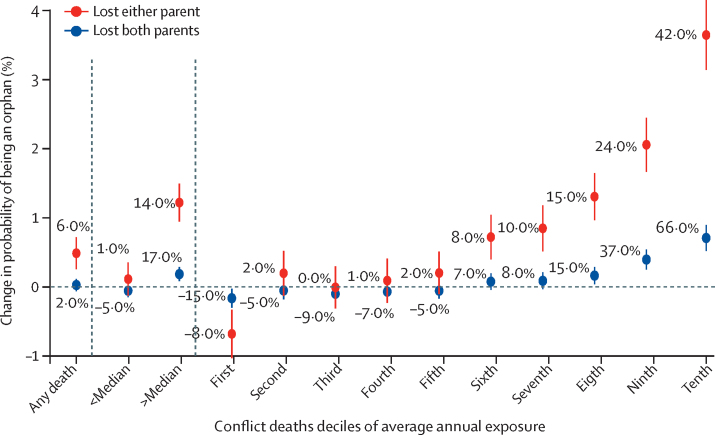
Change in the risk of being an orphan due to conflict The pooled exposure is based on any conflict, and the discretised exposure represents deciles of conflict intensity exposure as the average annual conflict-related deaths experienced by the child. The y-axis represents the increase in percentage points and the labels the average increase above the average prevalence of orphanhood for the entire sample. Error bars represent 95% CIs.

Among the 2·4 million children in our sample, the prevalence of children younger than 15 years who lost at least one parent was 204 276 (8·7%; children who lost both parents made up 1·1% of the sample; figure 3, table 2). The risk of orphanhood increased greatly with conflict above the median exposure (measured as average annual number of conflict-related deaths the child was exposed to). At the top decile of exposure, the risk of losing at least one parent increased by 3·4 percentage points (95% CI 3·1–4·2), a 42·0% (95% CI 36–48; figure 3) increase above the prevalence of orphanhood observed in the entire sample. The general patterns are similar for orphans who lost both parents, although the numbers are smaller (a 0·7 percentage points [0·5–0·9] increase in risk of two-parent orphanhood at the highest decile of conflict exposure, 66% [49–84] increase above baseline) and the pooled effect for all conflict exposures is indistinguishable from zero (0·03 [–0·06 to 0·12]; figure 3). On average, about a third of all orphans lost their mother, and two-thirds lost their father; however, conflict exposure increased the likelihood of losing both mothers and fathers (appendix p 13). At the top intensity decile, the probability of losing a father was 47% (95% CI 40–54) above baseline and the probability of losing a mother was 40% (30–50) above baseline.

**Figure 4:**
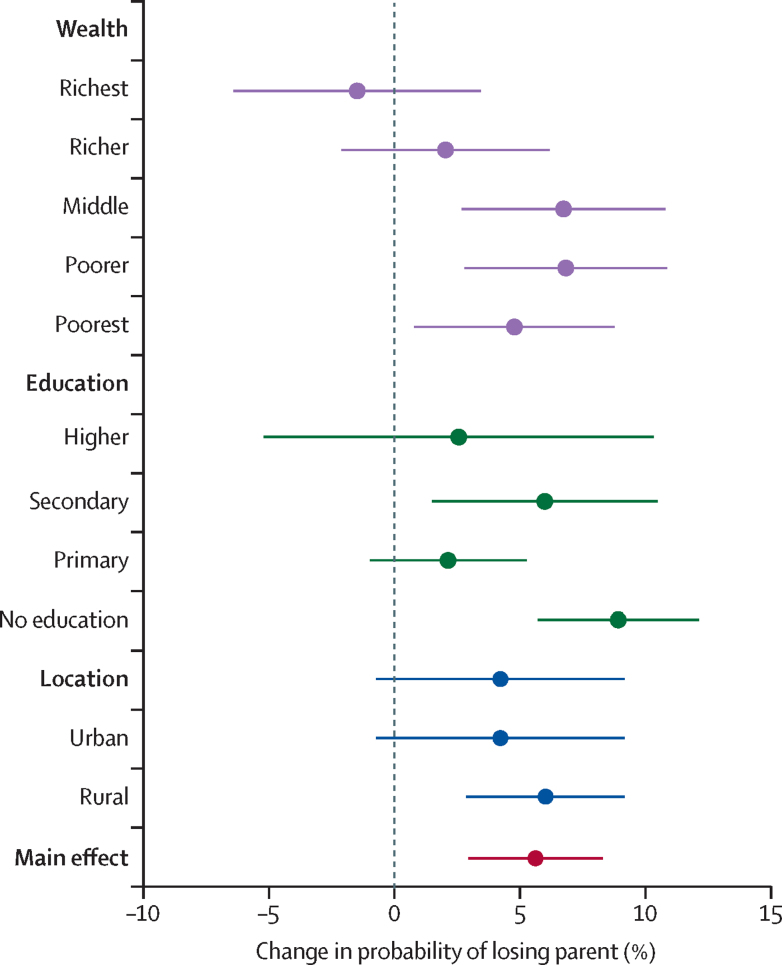
Heterogeneity in effect of conflict on orphanhood (any parent) Error bars indicate 95% CIs.

The heterogeneity of effects by household wealth, education of head of household, and place of residence (urban or rural) for any-parent orphans are shown in figure 4. Wealth and education appear protective for orphanhood. The effect of conflict on orphanhood was not statistically different between children living in rural and urban areas (p=0·55).

The sensitivity analysis of our findings to bias from population displacement showed that the net mortality effect of conflict on women of childbearing age remains positive under all sets of assumptions (appendix p 5). Even if we assume that 50% of the population is displaced (moves further than 50 km from the conflict), and mortality among those displaced is 50% lower than among those who stay, our overall mortality increase is lowered to 4–5% (and never becomes negative); however, under a more plausible scenario where 20% of the population is displaced and the mortality among the displaced women is 10–30% higher, we estimate an overall mortality increase of 24–28%. We also showed that our findings are robust to different fixed effect specifications (appendix p 2). Our leave-one-country-out analysis showed that our mortality results for women of childbearing age were most sensitive to the Rwanda genocide (appendix p 3). Without Rwanda in the sample, the increased mortality at the ninth and tenth deciles of exposure intensity declined from 47% to 9% and from 202% to 32%, respectively.

## Discussion

That armed conflicts have harmful population health effects, including deaths among women and orphanhood, is intuitive yet disputed.^34^, ^35^ We provided a detailed assessment of these effects, including their high variability based on the location, duration, and intensity of the conflicts. In this Article, we show that mortality among women of childbearing age increased by 21% (on average), and that the risk of orphanhood increased by nearly 6% following exposure to armed conflicts. Importantly, the mortality and orphanhood effects of armed conflict are statistically insignificant for low-intensity conflicts, but increased rapidly among women and children exposed to high-intensity conflicts. Although we observe a meaningful overall increase in mortality among women, the most important effects are associated with high-intensity conflicts, especially those in the highest decile of intensity, such as the Rwanda genocide, and the intense and chronic conflicts in Burundi, Liberia, Sierra Leone, and DR Congo.

Several patterns suggest reasons for the increased mortality. The close link with high-intensity conflicts supports the notion that women die as victims in intense fighting. However, other observations suggest that increases in mortality are associated with reasons indirectly related to combat. First, we find that women's mortality remains elevated for 1–3 years after the conflict ended (appendix p 11), which is more consistent with mortality increases beyond the acute conflict phase. Second, maternal mortality (about 10% of all conflict-related deaths we report) is increased in response to conflict, and might be more reflective of deteriorated health infrastructure than direct violence. However, the relative increases of maternal deaths in response to armed conflicts are smaller than the increases for all-cause deaths (11% *vs* 21%). The implication is that causes of death other than maternal mortality, especially violence-related mortality, are more responsive to conflict than maternal deaths. Maternal mortality might also be less responsive to conflict in part because of reduced fertility and fewer births in times of conflict.^36^, ^37^ Women are rarely combatants in armed conflicts, although this analysis suggests that, especially in intense conflicts, they are commonly victims. This work could help inform humanitarian efforts to mitigate the risks to women and children, primarily in relatively intense armed conflicts.

The similarity between the patterns of mortality among women and orphanhood suggests the possibility of a link between these events. The death of young women, if they had children, will leave orphans behind. Losing a parent has well documented consequences for health and human capital development of the child, and losing a mother is particularly harmful.^21^, ^38^ However, the link between orphanhood and conflict-related mortality is only partially assessed here because the death of fathers, in addition to mothers, leaves orphans behind (death among men was not examined in this study). Nevertheless, we note that 42% of the children in our sample were exposed to an armed conflict within 50 km of their home by the time they reached age 15 years (and over 20% exposed to conflicts of greater-than-median intensity). These estimates underscore the pervasiveness and magnitude of this issue.

The extent to which the underlying data enable consistent estimation of this study's outcomes deserves explicit discussion. The women whose survival records we measure do not necessarily reside with their sisters. In the extreme case where no sisters live close to one another, the true location of the sibling would be unrelated to her measured location. We would then be measuring the effect of noise, which is typically zero.^39^ In other words, the more measurement error we have in the women's location, the more we would expect our estimates to approach zero. Because the location of some women is probably measured with error, our effect sizes might be underestimated. Another source of measurement error is spatial imprecision from two sources. First, DHS displaces cluster locations by 2–5 km (and up to 10 km in 1% of rural clusters). Second, the coordinates provided represent the centroid of the DHS cluster, which might introduce additional measurement error to our exposure. A final source of measurement error is that the sister's time of death is measured annually, and we can only tell if conflict events happened in the same year as the sisters' deaths (ie, not if months before or after). Finally, we chose to use UCDP rather than an alternative repository of geolocated armed conflict (the armed conflict location event database) because of greater rigour in the definition of armed conflicts.^40^

Each conflict is unique, and the heterogeneity of conflict types and effect pathways is not completely captured by our disaggregation into intensity, chronicity, lagged effects, and personal features. The only defining feature of conflicts available in UCDP is whether the conflict is state based, one sided, or non-state. Because these features are of unclear significance to public health, and because individuals might be exposed to more than one type of conflict in any year (complicating attribution of effects), we did not consider these features for our analyses.

Both study outcomes are associated with potential bias from population displacement. This concern is important because the displaced population might be affected by conflict differently than the non-displaced population, and, therefore, might alter our estimation of conflict's overall health effects. Our simulations of the displaced population show that even extreme assumptions about displacement and mortality reductions among displaced people do not compensate for armed conflict's increased mortality risk for women. Future work could assess the role of conflict on mortality among men and improve estimates of conflict's effect on population displacement.

The health of women and children is a global priority, and in that respect quantifying the elevated risks and harms to women and children in areas of conflict is an important endeavour. Deaths of young women in sub-Saharan Africa are exceptionally high relative to developed countries, and the consequences of orphanhood—already high because of HIV—mean that the harmful effects of armed conflict extend to the next generation. Identifying effective approaches to prevent and mitigate the deleterious effects of armed conflicts on women and children should be a global priority.
