# Supplementary material for: Women and children living in areas of armed conflict in Africa: a geospatial analysis of mortality and orphanhood
Source: Lancet Glob Health. 2019 Oct 24;7(12):e1622–31. doi: 10.1016/S2214-109X(19)30407-3 (PMC7024993; doi:10.1016/S2214-109X(19)30407-3)
Supplement: Supplementary appendix [file mmc1.pdf]

THE LANCET
Global Health

Supplementary appendix

This appendix formed part of the original submission and has been peer reviewed. We post it as supplied by the authors.

Supplement to: Wagner Z, Heft-Neal S, Wise PH, et al. Women and children living in areas of armed conflict in Africa: a geospatial analysis of mortality and orphanhood. *Lancet Glob Health* 2019; published online Oct 24. http://dx.doi.org/10.1016/S2214-109X(19)30407-3.

WOMEN AND CHILDREN LIVING IN AREAS OF ARMED CONFLICT: A GEOSPATIAL ANALYSIS OF MORTALITY AND ORPHANHOOD IN AFRICA

[Supplementary Appendix]

Zachary Wagner, Sam Heft-Neal, Paul Wise, Robert E. Black, Marshall Burke, Ties Boerma, Zulfiqar A. Bhutta, Eran Bendavid

Table of Contents

1 Robustness of Main Results ... 2

1.1 Robustness to different levels of fixed effects ... 2

Figure S1. The impact of armed conflict within 50km on mortality of women of childbearing age using different levels of fixed-effects. ... 2

1.2 Robustness to leaving each country out individually ... 3

Figure S2. Sensitivity of estimates to leaving each country out of the sample ... 3

1.3 Sensitivity to excluding displaced migrants ... 4

Figures S3. Coefficient estimates of how conflict effects mortality for women of childbearing age under different assumptions about the displaced population ... 5

2 Methodology for deaths related to conflict ... 6

3 Regression Tables ... 7

Table S1. regression output for Figure 1A ... 7

Table S2. regression output for Figure 1B ... 8

Table S3. regression output for Figure 3 ... 9

4 Additional Analysis and Data ... 10

4.1 Impact of armed conflict for women of childbearing age across time ... 10

Figure S4. Impact of armed conflict on women's mortality over time ... 11

4.2 Impact of armed conflict on maternal mortality ... 12

Figure S5. Impact of armed conflict on maternal mortality ... 12

4.3 Impact of armed conflict on losing a mother or losing a father ... 13

Figure S6. Change in the risk of being an orphan in relationship to conflict (losing mother vs. losing father) 13

4.4 Additional Analyses ... 14

Table S4. Impact of conflict within 100km radius ... 14

Table S5. Number of deaths among women of childbearing age related to conflict for study countries (inputs for figure 2B) ... 15

1 Robustness of Main Results

1.1 Robustness to different levels of fixed effects

In the main text, we used DHS cluster fixed-effects and year fixed-effects. Although this helps to improve identification of the causal effect of conflict, such granular fixed effects restrict potentially important variation that occurs across time and space. Below, we test the sensitivity of our results to inclusion of less restrictive fixed-effects (Figure S1). All specifications imply that exposure to deadly conflict leads to meaningful increases mortality risk for women of childbearing age. The far right specification is the estimate reported in the main text and is slightly larger and less precise than the less restrictive specifications.

Figure S1. The impact of armed conflict within 50km on mortality of women of childbearing age using different levels of fixed-effects.

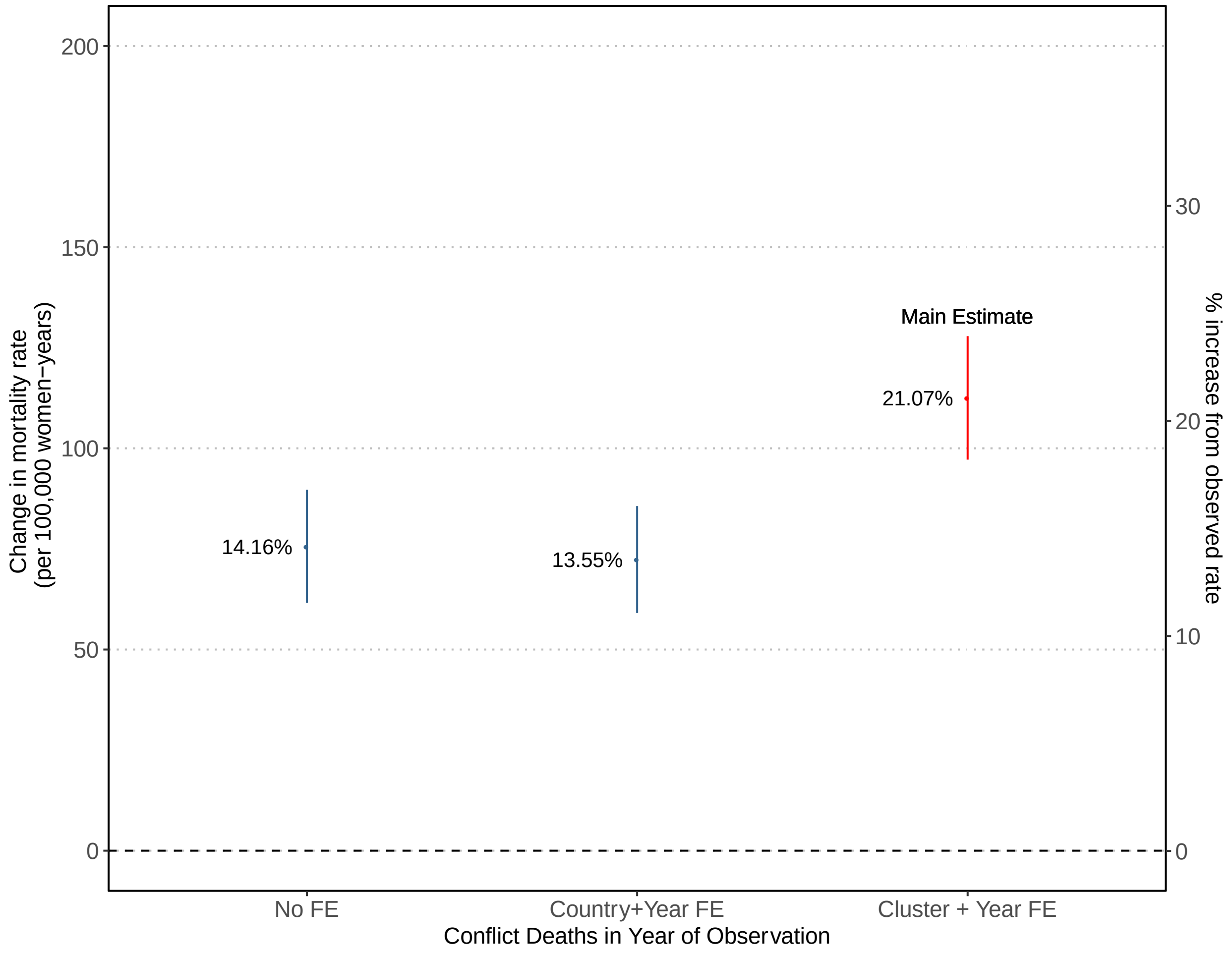

1.2 Robustness to leaving each country out individually

Below, we present the sensitivity of our estimates for the 9th intensity decile and 10th intensity decile to excluding one country's observations. The dashed line represents the estimate reported in the main text. Figure S2 demonstrates that our results highly sensitive to the inclusion of Rwanda, where particularly intense conflicts occurred. However, even after excluding Rwanda, the 9th and 10th deciles are sizable and significant – 46 and 166 deaths per 100,000 person years (9% and 32% increases).

Figure S2. Sensitivity of estimates to leaving each country out of the sample

Effect of conflicts in the 9th intensity decile	Effect of conflicts in the 10th intensity decile

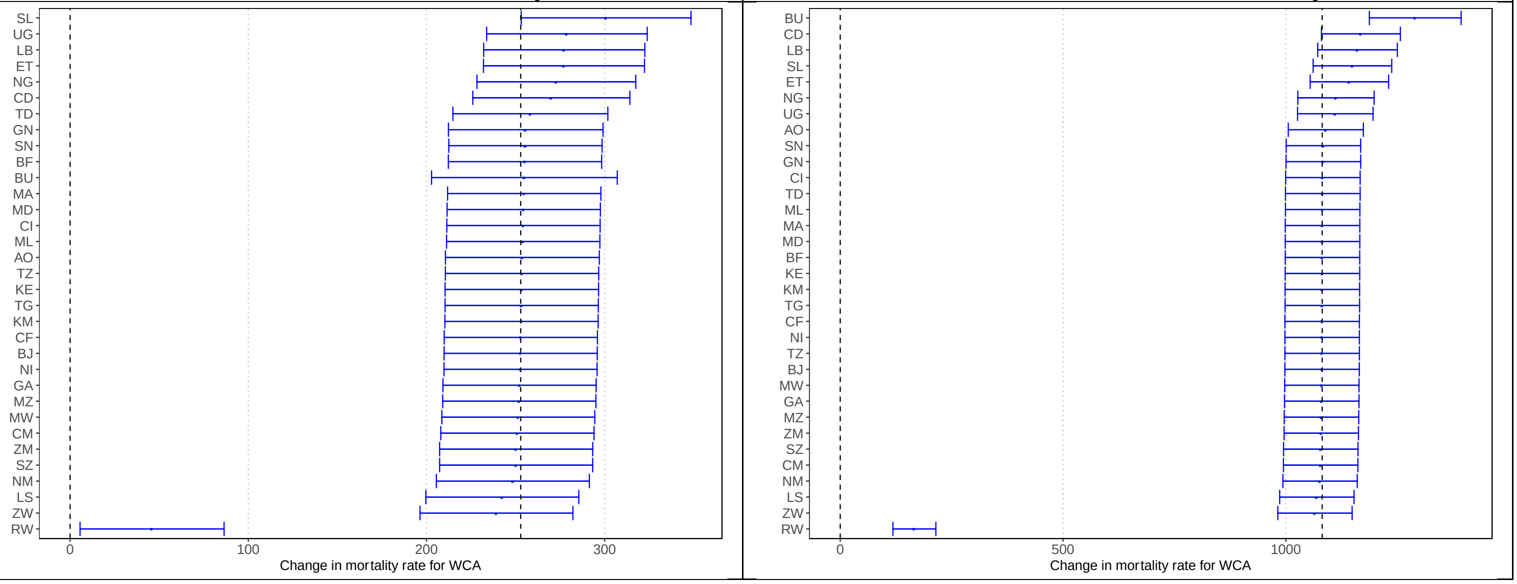

1.3 Sensitivity to excluding displaced migrants

Our primary analysis uses information provided in DHS surveys about the location of the cluster (roughly village or neighborhood) where the index women was interviewed. If the woman was displaced as a result of conflict such that she no longer resided within 50km of the conflict zone, then our conflict exposure measure would be misclassified. If the people who are displaced have systematically higher or lower mortality than those who stay (either higher or lower mortality are plausible: displacement is a risky event; and at the same time staying near conflict is also risky), then our main effects may be biased. In this section, we examine the sensitivity of our binary estimates for women of childbearing age (Figure 1 in the main text) to excluding potential migrants using a simulation exercise. We simulate the impact of assumptions about displacement along 2 dimensions: the size of the displaced population, and their relative mortality. We first assume a displacement rate and a difference in mortality rate among migrants relative to non-migrants. We then add synthetic migrants to our data set (in cluster-years that experienced conflict only) according to the assumed displacement rate (e.g., a cluster-year with 10 observations would be added an addition 2 observations if the displacement rate were 20%). We set our mortality variable for the synthetic migrants according to a binomial distribution with the assumed migrant probability of death as the success rate. We then re-estimate our main regression model with the new data set. We repeat this process for a range of displacement rates (10% to 50%) and a range of death rates (50% lower to 50% higher relative to the death rate of non-migrants). The steps below outline this procedure.

1. For each cluster-year where a conflict occurred we identify the mortality rate and the number of siblings. This creates a data set with 1 observation per cluster-year.
2. We duplicate cluster-year observations according to the displacement rate and the number of siblings per cluster-year. Cluster-year rows are duplicated such that the number of rows is equivalent to the displacement rate multiplied by the number of siblings in the cluster-year (with rounding). For example, if the displacement rate is 30% and the number of siblings is 10, this cluster would have three rows.
3. To impute whether simulated women in each cluster year died, we sample from a binomial distribution with probability of success set as the mortality rate in that cluster-year multiplied by 1 + d, where d is some proportional change in the mortality rate.
4. We append this new data set of simulated migrants to the original data and re-run our main regression.

We repeated this exercise 5 times for each combination of displacement rate and probability death (150 new estimates in total). Figure S3 plots the coefficients from this exercise. This figure demonstrates that even under extreme assumptions about the displaced population (e.g., 50% displaced and 50% lower mortality rate) our coefficient estimates are still positive, albeit relatively small. Under more realistic assumptions about migration (e.g., 20% displacement rate), then our estimates are still substantial (> 9% increase in mortality rate for WCA).

Figures S3. Coefficient estimates of how conflict effects mortality for women of childbearing age under different assumptions about the displaced population

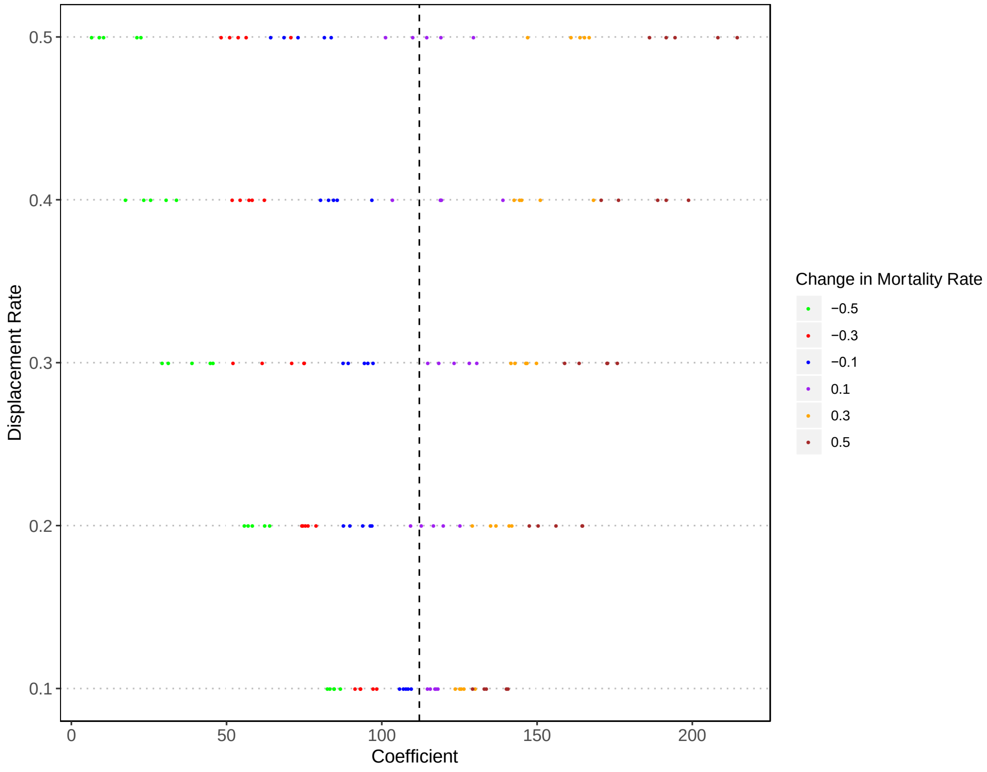

Each of the 150 points represents a different coefficient estimate from estimating eq. (1) in the main paper after adding simulated migrants. The x-axis is the assumed migration rate and the colors indicate the assumed difference in mortality rate of migrants relative to non-migrants. Each combination of displacement rate and mortality rate was sampled repeated 5 times. The dotted line represents our coefficient estimate from the main paper.

2 Methodology for deaths related to conflict

In this section, we outline the procedure we used to estimate the number of WCA deaths related to conflict. We carried out the following steps.

1. First, we geospatially linked population estimates of WCA to conflicts in our study countries from 2000 to 2017 at a 1km spatial resolution. For spatial patterns of WCA population estimates, we utilize 1km estimates aggregated to an 0.1x0.1 degree grid.[1] We spatially and temporally (yearly) linked these grid-level population estimates to the UCDP conflict data .
2. We then identified whether the population in each grid-cell-year was exposed to conflict within 50km and of what intensity (number of deaths). The result is a grid-cell level panel data set that indicates the WCA population and conflict exposure in the respective grid-cell from 2000-2017.
3. We used this data set combined with our estimates on how conflict impacts mortality risk to estimate the number of women who would not have died in the absence of conflict. We applied estimates of the effect of each decile of intensity from equation 2 and figure 1 in the main text. In other words, for each grid-cell-year, we multiplied the population estimate for the grid-cell-year by the coefficient that reflected the level of conflict exposure for that grid-cell-year (0 for no conflict exposure). We then took the sum of this measure across all years for each grid-cell. This allowed us to estimate the number of WCA deaths for each grid-cell-year and in each grid-cell from 2000-2017.
4. We then plotted the number of deaths for each grid-cell from 2000-2017 to create figure 2A and summed over all grid-cells for each country to create figure 2B.

The sum over all grid-cells-years gives the total number of WCA deaths related to conflict from 2000-2017 (310,494 in study countries and 426,558 if we extrapolate to all countries in Africa).

3 Regression Tables

Table S1. regression output for Figure 1A

	Mortality Risk (per 100,000 Person-Years		
	Binary	Above/Below Median Intensity	Intensity
	(1)	(2)	(3)
Any death	112.532***		
	(7.828)		
Below median#		9.524	
		(7.731)	
Above median#		271.506***	
		(13.153)	
1-2 deaths			-16.137
			(12.387)
3-4 deaths			2.919
			(18.134)
5-9 deaths			47.698***
			(14.509)
10-19 deaths			38.557**
			(15.262)
20-35 deaths			3.443
			(15.161)
36-64 deaths			42.714***
			(16.357)
65-136 deaths			45.806***
			(16.462)
137-341 deaths			175.919***
			(21.204)
342-825 deaths			252.829***
			(21.930)
826+ deaths			1,081.429***
			(42.648)
Observations	19,286,387		19,286,387
R^2	0.004		0.004

#The median number of deaths was 35
**p<0.05; ***p<0.01

Table S2. regression output for Figure 1B

Chronicity	Mortality Risk (per 100,000 Person-Years
First year only	39.379***
	(8.792)
2 years	81.473***
	(13.051)
3 years	225.661***
	(23.718)
4 years	336.589***
	(31.815)
5+ years	245.593***
	(20.389)
Observations	19,286,387
R^2	0.004

***p<0.01

Table S3. regression output for Figure 3

	Risk of having lost a parent			
	Both Parents		Either Parent	
	Binary	Intensity	Binary	Intensity
	(1)	(2)	(3)	(4)
Any death	0.0003		0.005^{***}	
	(0.0005)		(0.0010)	
Decile 1		-0.002^{**}		-0.007^{***}
		(0.0010)		(0.0020)
Decile 2		-0.001		0.002
		(0.0010)		(0.0020)
Decile 3		-0.001^{*}		-0.0001
		(0.0010)		(0.0020)
Decile 4		-0.001		0.001
		(0.0010)		(0.0020)
Decile 5		-0.001		0.002
		(0.0010)		(0.0020)
Decile 6		0.001		0.007^{***}
		(0.0010)		(0.0020)
Decile 7		0.001		0.008^{***}
		(0.0010)		(0.0020)
Decile 8		0.002^{**}		0.013^{***}
		(0.0010)		(0.0020)
Decile 9		0.004^{***}		0.021^{***}
		(0.0010)		(0.0020)
Decile 10		0.007^{***}		0.037^{***}
		(0.0010)		(0.0030)
Observations	2,354,041	2,354,041	2,354,041	2,354,041
R^2	0.054	0.054	0.104	0.104

$^{*}p<0.1$; $^{**}p<0.05$; $^{***}p<0.01$

4 Additional Analysis and Data

4.1 Impact of armed conflict for women of childbearing age across time

In prior work, we find that the negative effects of armed conflict for children lasts many years after the conflict even occurs.[2] To estimate the lingering effects of armed conflicts for women, we include lagged conflict exposure measures in our regression model. In the equation below the D_{lct}^{q} terms represent indicators for whether women i experienced conflict exposure in q years prior to the current year. Other terms are identical to equations 1 and 2 in the main text.

$$Women's\_death_{ilct} = \sum_{q=-5}^{0} \beta_q D_{lct}^{q} + \rho \boldsymbol{X}_{ilct} + \eta_{lc} + \gamma_t + \epsilon_{ilct}$$

Figure S4 plots the β_q terms, which represent the effect of armed conflict q years after the event took place. This figure shows that a conflict event resulted in increased mortality for women for two years (the year of the event and the following year).

Figure S4. Impact of armed conflict on women's mortality over time

Any death in year 0

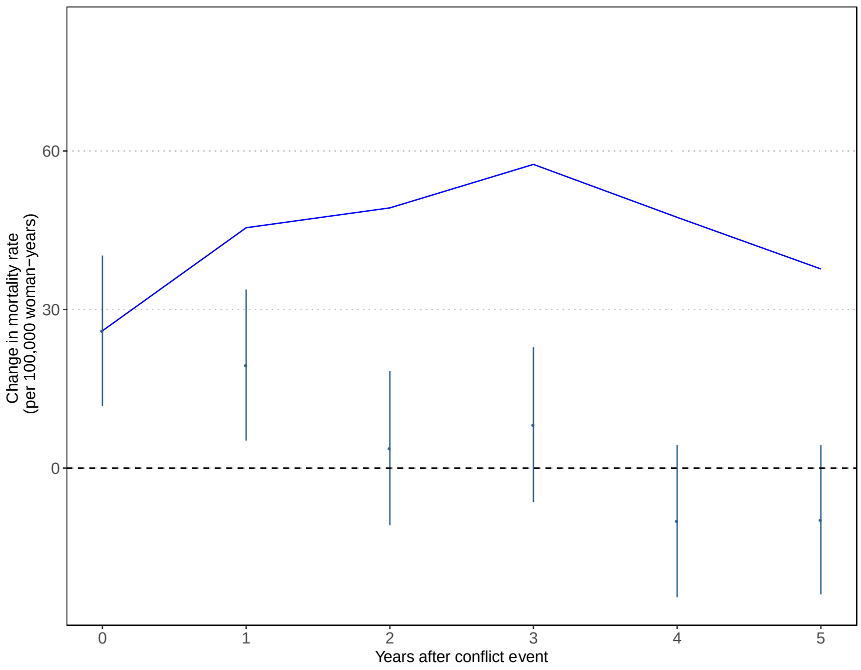

Above median intensity in year 0 (more than 35 deaths)

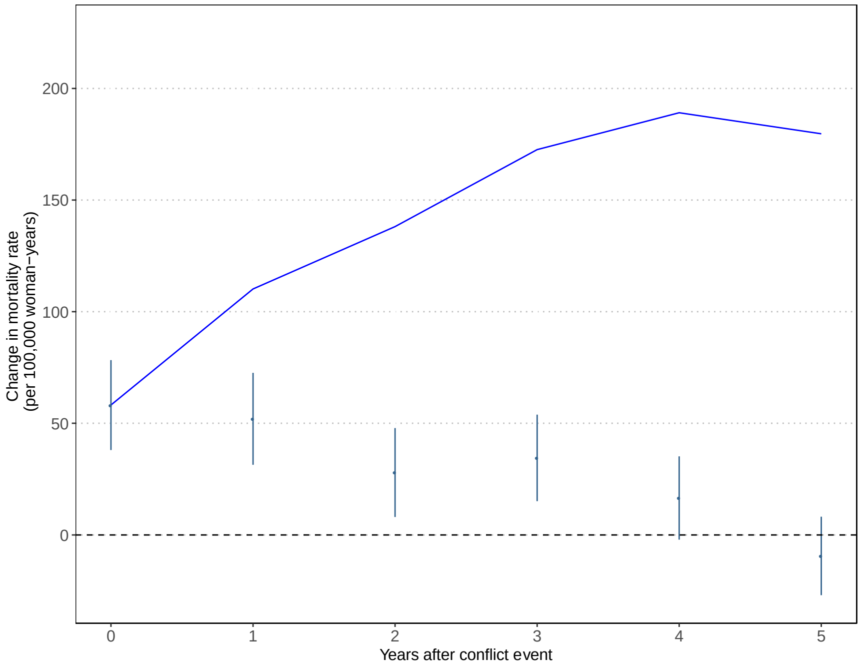

Year = 0 is the year in which the conflict occurred. Each point represents a coefficient from the same regression model.

4.2 Impact of armed conflict on maternal mortality

The only cause of death we observe is maternal mortality. We estimate the increase in maternal mortality following armed conflict by restricting our dependent variable to deaths directly related to pregnancy. Figure S5 includes these results.

Figure S5. Impact of armed conflict on maternal mortality

A. Intensity of conflict (deciles of conflict deaths)

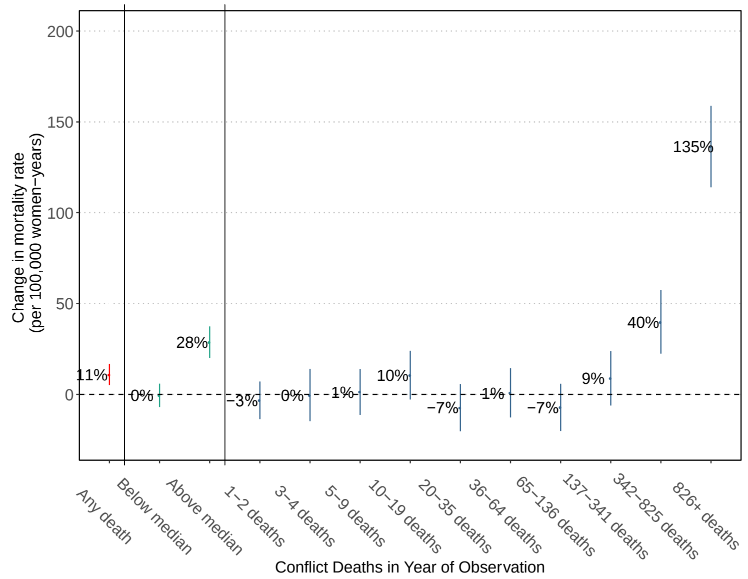

B. Chronicity of conflict (consecutive years of exposure)

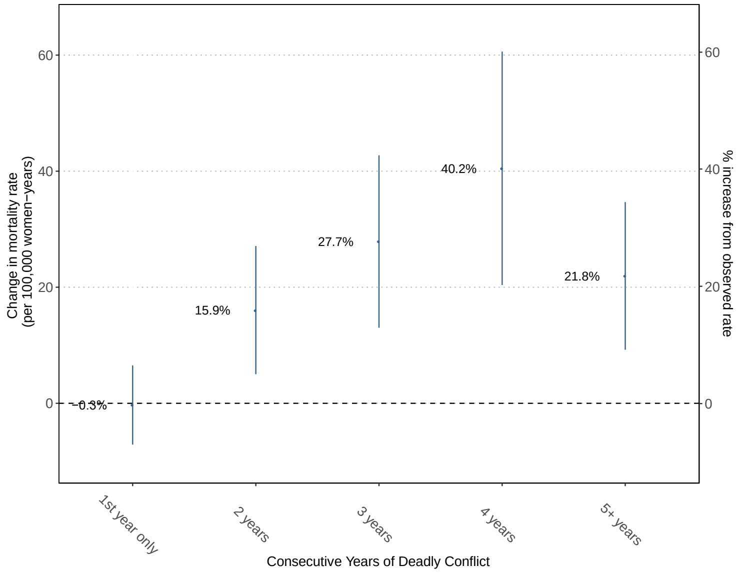

Change in the risk of maternal death for a woman of childbearing age as a function of exposure to conflict within 50km. In Figure 1A conflicts are designated as a binary exposure (Any death) or as deciles of exposure intensity by the number of direct combat-related deaths within 50km. In Figure 1B conflict intensity is measured by the number of consecutive years the index woman's cluster of residence has been exposed to nearby conflict (50km). Increase in risk of maternal death is measured in deaths per 100,000 women-years (left axis) and percent increase above the mean mortality of the entire sample (next to data points).

4.3 Impact of armed conflict on losing a mother or losing a father

Figure S6. Change in the risk of being an orphan in relationship to conflict (losing mother vs. losing father)

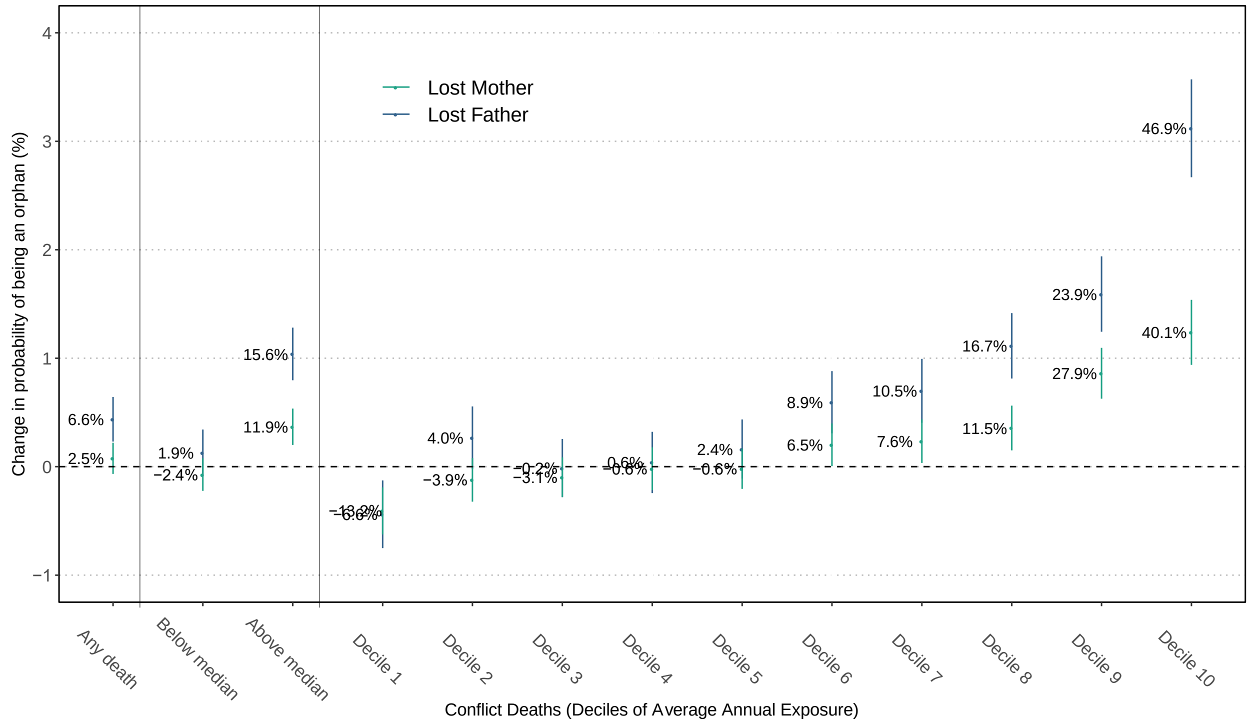

The top series represents the increased risk of losing a father, and the bottom series represents the increased risk of losing a mother. The pooled exposure (left-most estimates) is based on any conflict, and the discretized exposure represents deciles of conflict intensity exposure as the average annual conflict-related deaths experienced by the child. The y axis represents the increase in percentage points, and the labels represent the increase above the average prevalence of orphanhood for the entire sample.

4.4 Additional Analyses

Table S4. Impact of conflict within 100km radius

	Mortality Risk (per 100,000 Person-Years		
	Binary	Above/Below Median Intensity	Intensity
	(1)	(2)	(3)
Any death	(1.36)		
	(6.15)		
Below median#		-62.035***	
		(6.78)	
Above median#		94.309***	
		(8.94)	
1-2 deaths			-71.163***
			(11.23)
3-4 deaths			-73.719***
			(15.47)
5-9 deaths			-38.273***
			(12.78)
10-19 deaths			-43.509***
			(11.98)
20-35 deaths			-56.689***
			(12.23)
36-64 deaths			-61.817***
			(12.53)
65-136 deaths			(5.02)
			(13.46)
137-341 deaths			12.21
			(13.58)
342-825 deaths			142.481***
			(17.95)
826+ deaths			562.975***
			(23.14)
Observations	19,286,387	19,286,387	19,286,387
R^2	0.004		0.004

#The median number of deaths was 35
**p<0.05; ***p<0.01

Table S5. Number of deaths among women of childbearing age related to conflict for study countries (inputs for figure 2B)

Country	Deaths
Nigeria	106,819
Democratic Republic of the Congo	51,118
Burundi	37,008
Rwanda	19,312
Egypt	15,846
Uganda	12,295
Cote d'Ivoire	10,148
Ethiopia	10,016
Kenya	9,324
Liberia	6,624
Cameroon	6,072
Central African Republic	5,279
Guinea	4,130
Angola	3,536
Chad	2,922
Sierra Leone	2,830
Togo	1,303
Morocco	1,069
Ghana	920
Tanzania	844
Madagascar	645
Zimbabwe	496
Senegal	493
Mali	462
Niger	371
Mozambique	241
Burkina Faso	158
Benin	112
Zambia	80
Namibia	22
Gabon	0
Comoros	0
Lesotho	0
Malawi	0
Swaziland	0

1. WorldPop [Internet]. [accessed 2019 Mar 19];Available from: https://www.worldpop.org/

2. Wagner Z, Heft-Neal S, Bhutta ZA, Black RE, Burke M, Bendavid E. Armed conflict and child mortality in Africa: a geospatial analysis. The Lancet 2018;392(10150):857–65.
